# Supplementary material for: Mathematical Modeling of Remdesivir to Treat COVID-19: Can Dosing Be Optimized?
Source: Pharmaceutics. 2021 Jul 31;13(8):1181. doi: 10.3390/pharmaceutics13081181 (PMC8400702; doi:10.3390/pharmaceutics13081181)
Supplement: Supplementary file 1 [file pharmaceutics-13-01181-s001.zip › pharmaceutics-1279978-supplementary.pdf]

Supplementary Materials: Mathematical Modeling of Remdesivir to Treat COVID-19: Can Dosing Be Optimized?

Jessica M. Conway and Pia Abel zur Wiesch

Supplementary Figures and Table

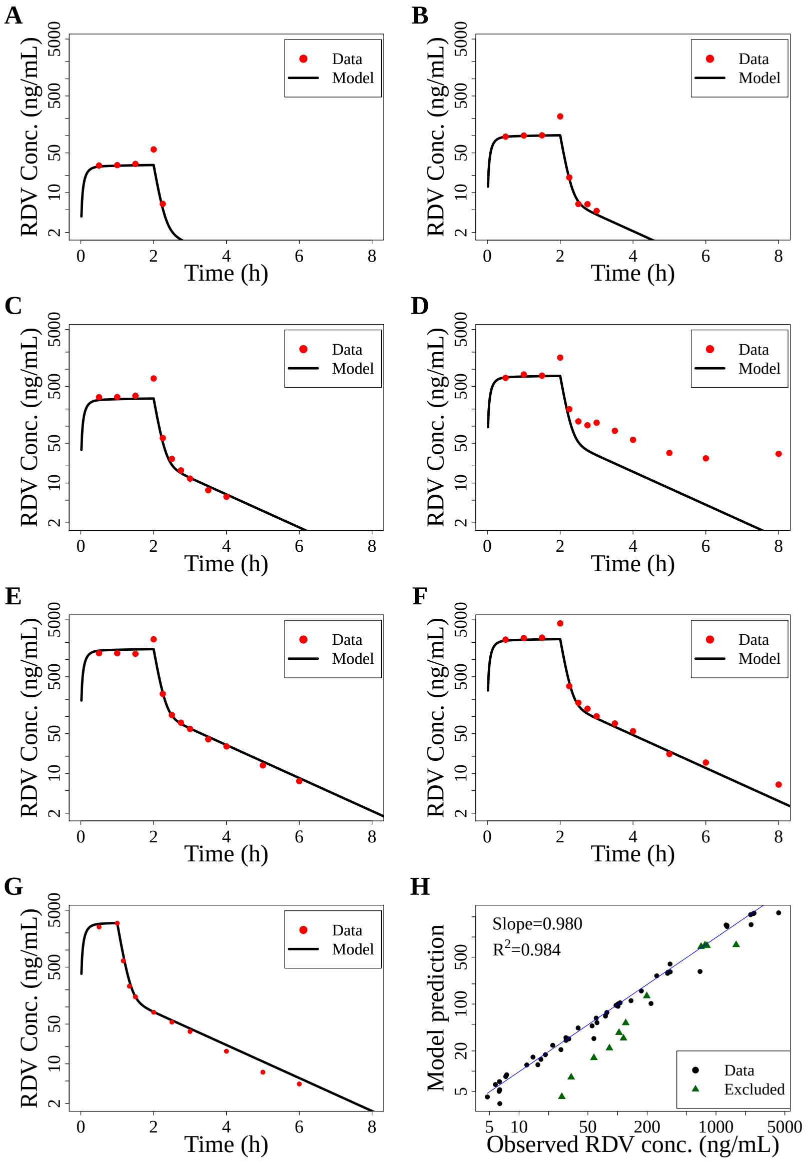

Figure S1. Median RDV plasma concentration from single dose experiments (Humeniuk et al. 2020 [1]), with different RDV dose sizes and infusion durations, compared to our pharmacokinetic model prediction (Equation (1), main text, also in Supplementary Text S1, below, as Equation S2). (**A**) 3 mg dose for a 2 h infusion; (**B**) 10 mg dose for a 2 h infusion; (**C**) 30 mg dose for a 2 h infusion; (**D**) 75 mg dose for a 2 h infusion; (**E**) 150 mg dose for a 2 h infusion; (**F**) 225 mg dose for a 2 h infusion; (**G**) 150 mg dose for a 1 h infusion (first administration of RDV from a multi-dose study). (**H**) Linear regression comparing the model predictions with the data to demonstrate how well the model explains the data. Data from 75 mg dosing excluded in the fitting are marked in green.

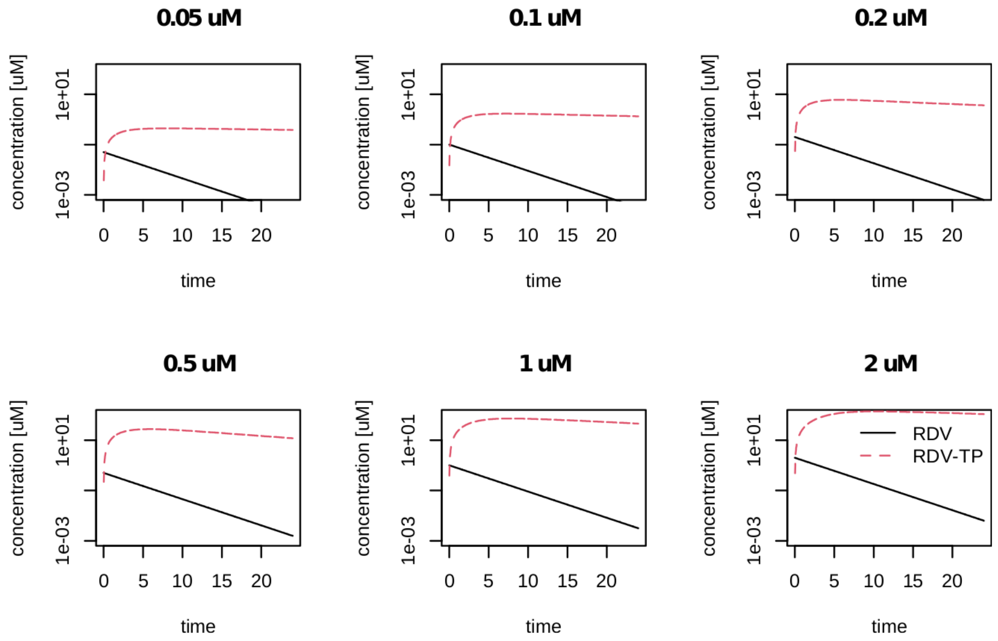

Figure S2. Extracellular RDV concentrations and intracellular RDV-TP concentrations during in vitro dose-response experiments. This figure shows the concentrations of RDV and RDV-TP when an initial RDV concentration is supplied in cell culture medium. The parameters are the same as in main text Figures 2(C,D). The title indicates the initial RDV concentration (in μM), the black lines the RDV concentration over time, the red lines the intracellular RDV-TP concentrations over time. We assume that the RDV concentration exponentially declines at 37°C as described in Avateneo et al. 2020 [2].

Table S1: Pharmacokinetic parameters for metabolite RDV-TP from single-dose experiments from Humeniuk et al. 2020 [1], with different RDV dose size and infusion durations, compared to our pharmacokinetic model prediction (Equation (1), main text, also in Supplementary Text S1, below, as Equation S2). Pharmacokinetic parameters are given in terms of mean and %CV, with the exception of the half-life $t_{1/2}$ for which uncertainty is explained via IQR. The model predictions extending beyond the %CV or IQR is italicized.

Dosing	Pharmacokinetic parameter (units)	Median (%CV or IQR)	Model prediction
75 mg infused over 2 hours	C_{max} (µM)	2.5 (16.2)	2.5
	C_{24} µM	2.2 (23.3)	1.67
	AUC_{∞} (hµM)	176 (31.1)	184
	$t_{1/2}$ (h)	42.7 (30.6-47.4)	*48.0*
150 mg infused over 2 hours	C_{max} (µM)	6.0 (46.1)	8.79
	C_{24} µM	3.7 (40.9)	4.0
	AUC_{∞} (hµM)	297 (28.3)	381
	$t_{1/2}$ (h)	36.0 (27.3-41.5)	34.4
75 mg infused over 30 minutes	C_{max} (µM)	5.9 (37.7)	5.5
	C_{24} µM	3.3 (55.7)	2.0
	AUC_{∞} (hµM)	394 (49.9)	291
	$t_{1/2}$ (h)	49.0 (26.6-69.5)	39.4

Supplementary Text (Text S1)

Details on development of pharmacokinetic model describing RDV and RDV-TP dynamics in plasma and associated parameter estimation, with the optimal dosing approximation calculation.

1. Pharmacokinetic model and parameter estimation

1.1. Model of remdesivir prodrug in plasma

We begin by estimating model parameters for remdesivir in plasma only. We consider extra-cellular concentration of RDV in plasma and the periphery, R and P.

$$\frac{dR}{dt} = \frac{q_0}{V_r} H(t-\tau) - (\beta + \delta_R)R + kP \tag{S1}$$
$$\frac{dP}{dt} = \beta R - (k + \delta_P)P$$

Note that we assume for this model a single dose, initiated at time 0, with duration τ, to compare with data in Humeniuk et al. (2020) [1]. We later append to this model a equation describing the active triphosphate metabolite, RDV-TP (GS-443902).

We fit this model to the longitudinal data in Figures 1a and 2a of Humeniuk et al. (2020) [1]. Specifically we fit the model to the median values. Since we cannot extract the error bars from the graphs (they overlap), we cannot weight the fitting algorithm appropriately. This poses a difficulty because the dynamics of 75mg over 2 hours (Fig 1a) don't follow the trends set by the other dosings, but the data points in the tail, when drug concentrations decay, have large or no error bars. We therefore fit the model including and excluding this data set. However, we keep it in our analysis of the fit for transparency (Figure S1). Note that the model explain the 75mg dosing data when drug concentrations are high, when the majority of the conversion to intracellular metabolite takes place (see below), and fails to explain reported median drug concentrations only when drug concentrations are low. For fitting, we minimize the sum-of-square difference between the model and the median RDV concentration values for 3mg, 10mg, 30mg, 150mg, and 225mg infused over two hours, and 150mg infused over 1 hour, using Optim in R, with the Nelder-Mead algorithm followed up by stochastic annealing (SANN) to increase confidence that we have a likely global minimum.

Parameter estimates are given in Table S2.

Table S2: RDV model parameter descriptions.

Parameter	Description	Units	Estimate
q_0	Total mass of RDV infused	mg	n/a
τ	Duration of infusion	h	n/a
V_r	Apparent volume for RDV in plasma	L	6.09
β	Plasma-to-periphery RDV concentration transition rate	h^{-1}	4.42
δ_R	Elimination rate in plasma	h^{-1}	4.16
k	Periphery-to-plasma RDV concentration transition rate	h^{-1}	0.13
δ_P	Elimination rate in periphery	h^{-1}	0.61

To demonstrate the goodness-of-fit, in Fig. S3 we show the model predictions plotted against the observations and show the linear regression. With slope and R^2 close to 1, we show excellent fit.

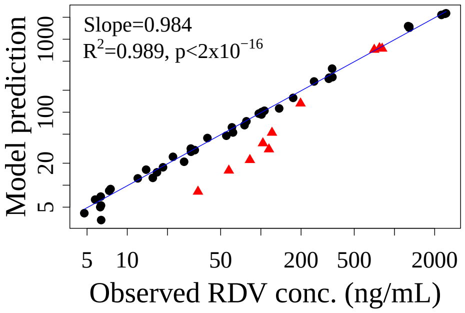

Figure S3. Model predictions vs observations on RDV concentrations for our model eq. (S1) for parameter estimates The 75mg data/prediction pairs are represented by the red triangles to show their outlier behavior. We show the linear regression between our model predictions and the data to increase confidence in our model. Note that the relationship is well explained by a linear relationship, with p-values<10^{-16} in both cases.

1.2. Model extension to predict dynamics of RDV-TP

The exact metabolic pathway that takes plasma RDV to the intracellular active triphosphate RDV-TP (GS-443902) is not yet confirmed [1,3,4]. However, there is consensus that intracellular RDV-TP is the active form of the drug [1,3,4]. We therefore model RDV and the RDV-TP only. The extended model is

$$
\begin{aligned}
\frac{dR}{dt} &= \frac{q_0}{V_r} H(t-\tau) - (\beta + \delta_R) R + kP - d\frac{R^2}{D^2 + R^2} \\
\frac{dP}{dt} &= \beta R - (k + \delta_P) P \\
\frac{dA}{dt} &= \sigma d \frac{R^2}{D^2 + R^2} - \mu_1 A - \mu_2 \frac{A^2}{M^2 + A^2}
\end{aligned}
\tag{S2}
$$

Here R represents plasma concentration of RDV, P concentration in the periphery, and A the intracellular RDV-TP concentration. In developing this model, we tested a variety of linear and nonlinear forms and alternative Hill parameters, finding this to best represent dynamics.

To our knowledge longitudinal data on RDV-TP is not available. To estimate parameters, we use parmacokinetic data provided Humeniuk et al. (2020) [1]. Instead of mixing data types for RDV and its active metabolites, we take parameters for RDV as given in Table S2, assuming that the amount that ultimately gets converted to the metabolite is negligible. We will verify this assumption. We then estimate remaining parameters using pharmacokinetic data in Table 4 of Humeniuk et al. (2020) [1], estimating on C_{max}, C_{24}, AUC_∞, and $t_{1/2}$ for intracellular RDV-TP following a 75 mg infusion over 30 minutes or 2 hours, or a 150 mg infusion over 2 hours. To generate a model-predicted AUC_∞, we integrate our result to 1000 days. To generate a model-predicted "terminal" $t_{1/2}$, we use the decay rate around $t = 48$ hours.

To estimate model parameters $\vec{\theta}$, we seek to minimize the error

$$
E(\vec{\theta}) = \sum_{j=1}^{3} \left[\frac{\left(\log(C_{max}) - \log(C_{max}^{pred})\right)^2}{IQR_{C_{max}}/\text{median}_{C_{max}}} + \frac{\left(\log(C_{24}) - \log(C_{24}^{pred})\right)^2}{IQR_{C_{24}}/\text{median}_{C_{24}}} \right.
$$
$$
\left. + \frac{\left(\log(AUC_\infty) - \log(AUC_\infty^{pred})\right)^2}{IQR_{AUC_\infty}/\text{median}_{AUC_\infty}} + \frac{\left(\log(t_{1/2}) - \log(t_{1/2}^{pred})\right)^2}{IQR_{t_{1/2}}/\text{median}_{t_{1/2}}} \right]
$$

where $j = 1, 2, 3$ correspond to dosings 75mg over 2 hours, 150mg over 2 hours, and 75 mg over 30 min, respectively. We use the log to balance the values, AUCs are so large they would otherwise dominate. Here we have used weights according to the ratio of the interquartile range (IQR) to the median as described in Arachchige et al. (2020) [5]. We use the IQR rather than the percent coefficient of variation (%CV) since the %CV is not provided for $t_{1/2}$ and when we attempt to derive one from the provided IQR assuming a normal distribution, we find that the normal distribution poorly explains the $t_{1/2}$. We estimate the IQR and median from the mean and %CV for C_{max}, C_{24}, AUC_{∞}, assuming a normal distribution.

To estimate parameters we minimize the eror using Optim in R, with the Nelder-Mead algorithm followed up by stochastic annealing (SANN) to increase confidence that we have a likely global minimum. Parameter estimates are available in Table S3.

Table S3: Metabolite model parameter descriptions.

Parameter	Description	Units	Estimate
d	Nonlinear plasma RDV to intracellular active triphosphate metabolite transport and activation rate	mg/L/h	31.57
D	RDV concentration yielding 50% of the maximal transport/activation rate	mg/L	3516.27
σ	Conversion of concentrations from mg/L to M (not estimated)	mol/mg	1
μ_1	Linear elimination rate of RDV-TP	h^{-1}	1.55
M	RDV-TP concentration at which the nonlinear elimination rate is 50% its maximum	μM	17.19
μ_2	Nonlinear metabolite clearance rate	μM/h	0.0084

We use our parameter estimates to predict C_{max}, C_{24}, AUC_{∞}, and $t_{1/2}$ for drug regimen in Table 4 of Humeniuk et al. [1], and compare with the data, using model eq. (S2) with parameter estimates deriving from either the weighted or unweighted fitting, given in Table S3. The results are shown in Table S1. All predictions but one land within the %CV of IQR of the data. The exception is the predicted half-life in the 75mg/2 hour dosing. In that case, the predicted half-life is just a hair outside the IQR: the prediction is 48 hours, the IQR is 30.6-47.4 hours. Note also that the model predictions lie within the %CV of reported metabolite C_{max}, C_{24}, AUC_{24}, in Table 16 of the EMA report (200mg dosing, we assume over 2 hours) (not shown).

Finally we verify that our model still predicts RDV dynamics consistent with data, that is, verify that our assumption that the conversion to RDV-TP is negligible relative to prodrug concentrations. Main text Fig. 1 and Fig. S1 show the full model predictions of RDV concentrations. We compare Fig. S3, showing model prediction vs data without active metabolite eq. (S1), with main text Fig. 1(C)/Fig. S1(H), showing the same in the model with active metabolite eq. (S2) and found little change.

2. Predicted optimal remdesivir dosing calculation

Using our nonlinear model eq. (S2), we can predict an RDV dosing rate that would maximize the intracellular active metabolite RDV-TP, thereby maximizing efficacy of RDV. Since the model eq. (S2) is nonlinear, the answer is not to simply maximize the prodrug plasma concentration.

We note from the data [1] (main text Fig. 1, supporting Fig. S1) that plasma RDV achieves steady-state rapidly after initiating the infusion. We can compute that that

steady-state, from our RDV model assuming constant drug infusion by solving simultaneously the equations

$$0 = \frac{r}{V_r} - (\beta + \delta_R)\bar{R} + k\bar{P} - d\frac{\bar{R}^2}{D^2 + \bar{R}^2} \tag{S3}$$

$$0 = \beta\bar{R} - (k + \delta_P)\bar{P} \tag{S4}$$

where r is the optimal dosing rate. We aim to maximize the amount of remdesivir ultimately converted to RDV-TP, which we approximate as the product of the conversion rate at the steady state $\bar{R}$, $d\bar{R}/(D^2 + \bar{R}^2)$, and the infusion duration τ. We are neglecting here RDV dynamics following end of infusion, but we observe from the data and our model prediction [1] (Fig. 1, Fig. S1) that that quantity is orders of magnitude smaller. That is, we maximize $d\bar{R}/(D^2 + \bar{R}^2)$, where $\bar{R}$ is the solution of eqs. (S3,S4), with respect to dosing rate r.

Thus we estimate that $r \approx 168$ mg per hour maximizes the intracellular active metabolite. That is, if we plan a 1 hour infusion, a total dose of approximately 168 mg should maximize the drug efficacy; for a 2 hour infusion, a total dose of approximately 336 mg maximizes the drug efficacy; and over half an hour, a total dose of approximately 84 mg would achieve the same aim.

References

1. Humeniuk, R.; Mathias, A.; Cao, H.; Osinusi, A.; Shen, G.; Chng, E.; Ling, J.; Vu, A.; German, P. Safety, Tolerability, and Pharmacokinetics of Remdesivir, An Antiviral for Treatment of COVID-19, in Healthy Subjects. *Clin Transl Sci* **2020**, *13*, 896–906. doi:10.1111/cts.12840.
2. Avataneo, V.; de Nicolò, A.; Cusato, J.; Antonucci, M.; Manca, A.; Palermiti, A.; Waitt, C.; Walimbwa, S.; Lamorde, M.; di Perri, G.; D'Avolio, A. Development and validation of a UHPLC-MS/MS method for quantification of the prodrug remdesivir and its metabolite GS-441524: a tool for clinical pharmacokinetics of SARS-CoV-2/COVID-19 and Ebola virus disease. *J Antimicrob Chemother* **2020**, *75*, 1772–1777. doi:10.1093/JAC/DKAA152.
3. Eastman, R.T.; Roth, J.S.; Brimacombe, K.R.; Simenov, A.; Shen, M.; Patnaik, S.; Hall, M.D. Remdesivir: A Review of Its Discovery and Development Leading to Emergency Use Authorization for Treatment of COVID-19. *ACS Cent Sci* **2020**, *6*, 672–683. doi:10.1021/acscentsci.0c00489.
4. Xu, Y.; Barauskas, O.; Kim, C.; Babusis, D.; Murakami, E.; Kornyeyev, D.; Lee, G.; Stepan, G.; Perron, M.; Bannister, R.; Schultz, B.E.; Sakowicz, R.; Porter, D.; Cihlar, T.; Feng, J.Y. Off-Target *In Vitro* Profiling Demonstrates that Remdesivir Is a Highly Selective Antiviral Agent. *Antimicrob Agents Chemother* **2021**, *65*, e02237–20. doi:10.1128/AAC.02237-20.
5. Arachchige, C.N.; Prendergast, L.A.; Staudte, R.G. Robust analogs to the coefficient of variation. *J Appl Stat* **2020**, *0*, 1–23. doi:10.1080/02664763.2020.1808599.
